# Associations of birth size, infancy, and childhood growth with intelligence quotient at 5 years of age: a Danish cohort study

**DOI:** 10.1093/ajcn/nqaa051

**Published:** 2020-03-31

**Authors:** Helene Kirkegaard, Sören Möller, Chunsen Wu, Jonas Häggström, Sjurdur Frodi Olsen, Jørn Olsen, Ellen Aagaard Nohr

**Affiliations:** Research Unit of Obstetrics and Gynecology, Department of Clinical Research, University of Southern Denmark, Odense, Denmark; Open Patient Data Explorative Network (OPEN), Department of Clinical Research, University of Southern Denmark and Odense University Hospital, Odense, Denmark; Open Patient Data Explorative Network (OPEN), Department of Clinical Research, University of Southern Denmark and Odense University Hospital, Odense, Denmark; Research Unit of Obstetrics and Gynecology, Department of Clinical Research, University of Southern Denmark, Odense, Denmark; MTEK Sciences Inc, Vancouver, British Columbia, Canada; Department of Epidemiology Research, Statens Serum Institute, Copenhagen, Denmark; Department of Clinical Epidemiology, Aarhus University Hospital, Aarhus, Denmark; Research Unit of Obstetrics and Gynecology, Department of Clinical Research, University of Southern Denmark, Odense, Denmark

**Keywords:** prenatal, infancy, childhood, growth, intelligence quotient, path analyses

## Abstract

**Background:**

The correlates of prenatal and postnatal growth on Intelligence Quotient (IQ) in childhood in term-born children living in high-income countries are not well known.

**Objectives:**

We examined how birth size and growth in infancy and childhood were associated with IQ at age 5 y in term-born children using path analysis.

**Methods:**

The study sample comprised 1719 children from the Danish National Birth Cohort who participated in a substudy in which psychologists assessed IQ using the Wechsler Primary and Preschool Scales of Intelligence–Revised. Measured weight, length/height, and head circumference at birth, 5 mo, 12 mo, and 5 y were included in a path model to estimate their total, indirect, and direct effects on IQ. All growth measures were included in the model as sex- and age-standardized *z*-scores.

**Results:**

After adjusting for potential confounders, a positive association between birth weight and IQ was observed, and 88% of the association was direct. Weight gain in infancy was associated with IQ [per *z*-score increase from 5 to 12 mo, IQ increased by 1.53 (95% CI: 0.14; 2.92) points] whereas weight gain from 12 mo to 5 y was not associated with IQ. Height and head circumference growth in childhood was associated with IQ [per *z*-score increase from 12 mo to 5 y, IQ increased by 0.98 (95% CI: 0.17; 1.79) and 2.09 (95% CI: 0.78; 3.41) points, respectively].

**Conclusions:**

In children born at term in an affluent country with free access to health care, higher IQ was seen with greater size at birth and greater weight gain in infancy. Also, greater growth in height and head circumference throughout the first 5 y of life was associated with higher childhood IQ whereas greater weight gain after the first year of life was not.

## Introduction

Nutrition is needed for growth including development of the brain (1), and studies have reported an association between growth of the fetus, indicated by birth size, and cognitive abilities in childhood (2), young adulthood, and in midlife (3, 4). Being born with low birth weight or preterm has been associated with lower intelligence quotient (IQ) in childhood (5, 6). For these children and children born small for gestational age, a postnatal catch-up growth in weight, length, and head circumference seems important to limit the reduction in cognitive ability (7–12). The influence of birth size as well as growth in infancy and childhood on IQ in healthy children born at term is less established and findings are in conflict. Some researchers examined only a specific segment of growth, for example, birth size or neonatal growth, and found a positive association that can attenuate in later childhood (13–16). Others examined birth size as well as postnatal growth throughout childhood; of these, some observed a positive association of all periods (17–19), others only for infancy growth (19, 20), and some observed no association (21). To understand the relative importance of both prenatal and postnatal growth, statistical analyses that consider the potential colinearity between included growth measures are needed. Some studies have used conditional analyses to account for previous growth measures (17, 19, 20). However, path analysis is a method to further disentangle direct from indirect effects of growth at different time points and can thus give a more varied insight into the associations of birth size, and infancy and childhood growth on IQ in childhood (22). Thus, the aim of the present study was to investigate how birth size and growth in infancy and childhood are associated with childhood IQ at age 5 y in children born at term. Furthermore, we aimed to distinguish between direct associations of birth size and infancy growth and indirect associations mediated through infancy and childhood growth.

## Methods

### Danish National Birth Cohort

This study was based on data from the Lifestyle During Pregnancy Study (LDPS) nested within the Danish National Birth Cohort (DNBC), which has been described in detail elsewhere (23, 24). Briefly, 91,381 women and their 100,413 pregnancies were included in the DNBC between 1996 and 2002. Women were recruited at their first antenatal visit to their general practitioner (GP); they were eligible if they planned to carry their pregnancy to term and spoke Danish well enough to take part in a personal interview. All women enrolled agreed to participate in 4 telephone interviews carried out approximately at weeks 16 and 31 of gestation and 6 and 18 mo postpartum. An FFQ was filled out at approximately week 26 of gestation, covering the previous month's dietary intake. The LDPS is a follow-up study of a subsample of the cohort, conducted between 2003 and 2006 when the children turned 5 y of age. The LDPS focused on prenatal lifestyle factors (primarily maternal alcohol exposure) and later neurodevelopment of the child, and the sampling was stratified on alcohol exposure. Exclusion criteria were mother's or child's inability to speak Danish, impaired hearing or vision of the child to the extent that a planned test session could not be performed, being a twin, or diagnosed with congenital disease prone to cause mental impairment (e.g., trisomy 21). A total of 3478 women were invited to participate in the LDPS. Of those invited, 1782 (51.0%) participated in a comprehensive follow-up assessment (24, 25). Further, when the child turned 7 y, a follow-up questionnaire was sent to all parents in the DNBC collecting information on current early childhood health (26).

The DNBC was approved by the Scientific Ethics Committee in Denmark and the Danish Data Protection Agency, and the latter also approved the present substudy. All the participants in the DNBC provided written informed consent.

### Growth measures

Birth weight, length, and head circumference measures were obtained from the Medical Birth Registry (MBR) covering all births in Denmark. All these measures were carried out by midwives before leaving the delivery room. At the second postpartum interview, ∼18 mo after birth, the mothers reported the child's weight, length, and head circumference measured at routine check-ups by their GP when the child turned 5 mo and 12 mo old as well as the exact dates of the measurements. The GPs followed national standards for measuring weight and length. Length was measured in a supine position to the nearest half centimeter, and weight was measured with 1 decimal place on a validated scale. The GP reported this information in the child's personal book, a health record kept by the parents and used to communicate between them, health professionals visiting the family at home, and the GP. At the 5-y LDPS follow-up, the research staff measured growth parameters of the child (head circumference, height, and weight) once, with the children wearing no shoes and light clothes. To increase comparability with other populations, we used the UK-WHO growth reference to calculate standardized sex- and age-specific *z*-scores for weight, length, and head circumference (27). To limit the variation in the time of the measurement for each fixed time point (5 and 12 mo), measurements performed before 3 mo or later than 7 mo for the 5-mo measurement were set to missing; similarly, measurements performed before 9 mo or later than 15 mo for the 12-mo measurements were regarded as missing. This was the case for only 1–1.5% of the measurements at each time point. We defined growth from birth to 5 mo as early infancy, growth from 5 to 12 mo as late infancy, and growth from 12 mo to 5 y as childhood.

### Outcome

Cognitive function as estimated by IQ was assessed as part of a 3-h neuropsychological assessment conducted by 10 trained psychologists following standardized test procedures (24) using the Wechsler Primary and Preschool Scales of Intelligence–Revised (WPPSI-R) (28). The full WPPSI-R comprises 5 verbal and 5 performance (nonverbal) subtests. To reduce the length of the test session, 3 verbal (arithmetic, information, and vocabulary) and 3 performance subtests (block design, geometric design, and object assembly) were used. Standard procedures were used to prorate full-scale IQ from this shortened form of the test when ≥2 verbal and ≥2 performance subtests were available. A Dutch study in children aged 4–7 y has shown high correlation between short forms of the WPPSI-III test and the original full version (29). No Danish WPPSI-R norms were available at the time of the study, and consequently Swedish norms were used to derive scaled scores and IQs (30). Thus, in this sample the theoretical designed IQ distribution of a mean of 100 and an SD of 15 is not expected and was not seen.

### Covariates

Potential covariates were chosen a priori based on the existing literature and path diagram of the path model. Included covariates are presented in **Supplemental Figure 1**. From the MBR, we obtained information on gender, maternal age at conception, birth order, and gestational age at birth. Information on socio-occupational status, maternal prepregnancy BMI as well as leisure-time exercise came from the first pregnancy interview. Socio-occupational status was categorized as high (≥4 y of education after high school, or job as manager), middle (skilled manual work, office, or service work), or low (unskilled work or unemployment) (31). Prepregnancy BMI (kg/m^2^) was estimated based on self-reported weight and height, and exercise in pregnancy was categorized as no exercise, 1–180 min/wk, or >180 min/wk. The FFQ information was used to characterize Western, intermediate, or health-conscious dietary patterns during pregnancy (32). Smoking status during pregnancy was obtained from the first pregnancy interview and the first postpartum interview, which provided information on smoking status during the last part of the pregnancy. Smoking during pregnancy was categorized as nonsmoking, smoking cessation at any time point, or smoking. Smoking status postpartum was obtained from the first postpartum interview and categorized as nonsmoking or smoking. The first postpartum interview also provided information on age when solid food was introduced, and divided into <4.0 mo, 4.0–5.0 mo, or >5.0 mo of age. In Denmark it is recommended to introduce solid food after the age of 4 mo and no later than the age of 6 mo; this 2-mo period we divided into 2 categories. Duration of any breastfeeding was obtained from both postpartum interviews and based on the age of the child when the mother stopped breastfeeding. It was divided into <20, 20–40, and >40 wk according to the 25th and 75th percentiles. In the second postpartum interview (18 mo postpartum), the mother gave information about the father's weight and height and we estimated his BMI. From the 5-y LDPS follow-up we obtained information on maternal IQ, which was based on the mean of 2 verbal subtests (information and vocabulary) from the Wechsler Adult Intelligence Scale (33) and Raven's Standard Progressive Matrices (34). Raw scores of each test were standardized based on the results from the full sample and weighted equally in a combined score which was restandardized to an IQ scale with a mean of 100 and an SD of 15. Finally, information on when the child started daycare was obtained from the 7-y follow-up study and divided into <8.0 mo, 8.0–14.0 mo, and >14.0 mo of age.

### Study population

**FIGURE 1:**
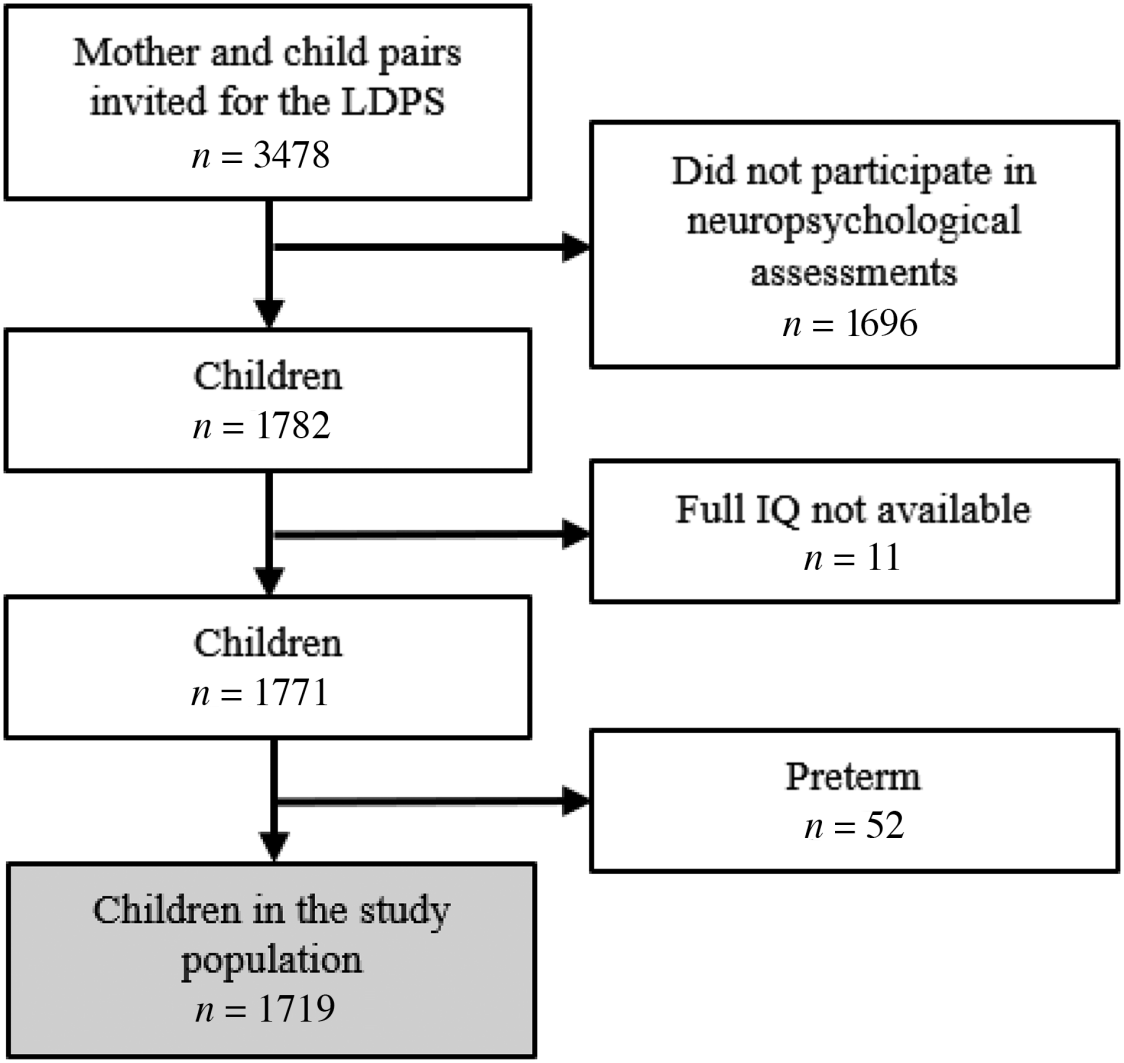
Flowchart of the study population. LDPS, Lifestyle During Pregnancy Study; IQ, intelligence quotient.

Of the 1782 children who participated in the neuropsychological assessments, the full IQ score was available for 1771 of the children; 97% of these were born at term, thus the study population encompassed 1719 children (**Figure 1**). There were no substantial differences between the participants and nonparticipants in the LDPS with regard to maternal age, parity, BMI, marital status, child sex, birthweight, or gestational age at birth (35). Children born small for gestational age or with low birth weight (<2500 g) (*n* = 8) were kept in the study population to examine the whole birth size distribution in children born at term.

### Statistical analyses

The distribution of offspring, maternal, and paternal characteristics was estimated as counts and proportions or means with SDs. To investigate the association between birth size and growth from birth to 5 y of age (measured as sex- and age-standardized *z*-scores of weight, length/height, and head circumference) and IQ, we used path analysis, a subset of structural equation modeling. Path analysis is an extension of regression analysis that simultaneously estimates the linear associations between all path variables and makes it possible to assess the total, direct, and indirect effects of each path variable (22, 36). The word “effect” is standard terminology for path analysis, but here it reflects a statistical association and not necessarily a causal effect. All growth measures were adjusted for preceding growth measures, and in the present study, the direct effect of a variable is the part of its effect not mediated through available intermediate growth measures, whereas the indirect effect is the effect mediated through these variables. The total effect is the sum of the direct and indirect effect. Per definition, the associations for the last growth measure will all be direct. Because all growth measures were conditioned on previous growth measures, the interpretation of, for example, 1 *z*-score higher weight-for-age at 12 mo will be a greater gain in weight from 5 to 12 mo resulting in 1 *z*-score higher weight-for-age at 12 mo.

Although some participants had missing information on some variables, they were all included in the analyses and contributed with all available information by using the estimation by maximum likelihood with missing values method (37). This modeling relies on the assumption that missing data are missing at random, conditional on the available data, and is expected to produce less bias than complete-case analysis (38). The residuals from the path analysis regressions were graphically examined for approximate consistency with the normal distribution assumptions and no strong violation was observed (**Supplemental Figure 2**).

All statistical analyses were weighted by sampling fractions because higher alcohol categories were oversampled in the LDPS. Furthermore, in model A, we adjusted for the following covariates at relevant regressions in our path analysis (see Supplemental Figure 1): sex, birth order, gestational age and maternal age, socio-occupational status, prepregnancy BMI, smoking, exercise, dietary intake during pregnancy, postpartum smoking, age when the child started in daycare, duration of any breastfeeding, time of introduction to solid food, paternal BMI 18 mo postpartum, and maternal IQ. In model B, we further adjusted the analyses of weight-for-length/height at the same point in time and at previous time points because greater weight for a given length/height could be a proxy of adiposity. Similarly, the analyses of length/height were adjusted for weight and analyses of head circumference were adjusted for weight and length/height. This was done because each measure might be an indicator of different underlying growth processes. We also performed analyses adjusted only for sex and gestational age, which are presented in **Supplemental Table 1**.

Finally, we generated restricted cubic splines (5 knots at the 5, 27.5, 50, 72.5, and 95 percentiles recommended for restricted cubic splines) to describe any significant associations between growth and childhood IQ in more detail by allowing a nonlinear association. In contrast to the path analyses, these analyses do not cope with missing data and multiple imputation was therefore used. Fifty copies of the dataset were generated, each of which had its missing values imputed by chained equations (39, 40). Variables with complete data (sex, birth order, gestational age, maternal age, IQ, and the sampling fraction weight) were included as additional explanatory variables in the imputation step. The splines are presented for a child with study population–specific reference values for categorical variables (marked in **Table1** with superscript 1) and means for continuous variables (maternal age, BMI, IQ, paternal BMI, gestational age).

**TABLE 1:** Characteristics of the study population

	*n* = 1719		
	*n*/mean	Percentage/±SD	Missing
Maternal characteristics			
Maternal socio-occupational status			6
High^1^	1041	61	
Middle	542	32	
Low	130	8	
Maternal age at conception, y	30.09	±4.41	0
Maternal prepregnancy BMI, kg/m^2^			33
<18.5	59	3	
18.5–24.99	1192	71	
25–29.99	315	19	
≥30	120	7	
Smoking during pregnancy			283
Nonsmoking^1^	969	67	
Smoking cessation	225	16	
Smoking	242	17	
Leisure-time exercise during pregnancy, min/wk			5
None^1^	1015	59	
1–180	547	32	
>180	152	9	
Dietary intake			350
Western	248	18	
Intermediate^1^	892	65	
Health-conscious	229	17	
Alcohol intake during pregnancy, units/wk			0
0	817	48	
>0–1	421	24	
>1	481	28	
Smoking in the first 6 mo postpartum			283
Nonsmoking^1^	1108	77	
Smoking	328	23	
Maternal IQ, points			2
<90	408	24	
90–110	873	51	
>110	436	25	
Paternal characteristics			
Paternal BMI at 18 mo postpartum, kg/m^2^			395
<18.5	6	0	
18.5–24.99	720	54	
25–29.99	509	38	
≥30	89	7	
Offspring characteristics			
Sex			0
Boys^1^	888	52	
Girls	831	48	
Gestational age, d	281.4	±8.8	0
Birthweight, g	3630.65	±486.09	12
Length at birth, cm	52.49	±2.15	19
Head circumference at birth, cm	35.38	±1.58	41
Birth order			0
First^1^	887	52	
Second	545	32	
Third or more	287	17	
Total breastfeeding duration, wk			385
<20	334	25	
20–40^1^	602	45	
>40	398	30	
Age when introduced to solid food, mo			284
<4.0	103	7	
4.0–5.0^1^	1089	76	
>5.0	243	17	
Age when daycare started, mo			469
<8.0	290	23	
8.0–14.0^1^	733	59	
>14.0	227	18	

**FIGURE 2:**
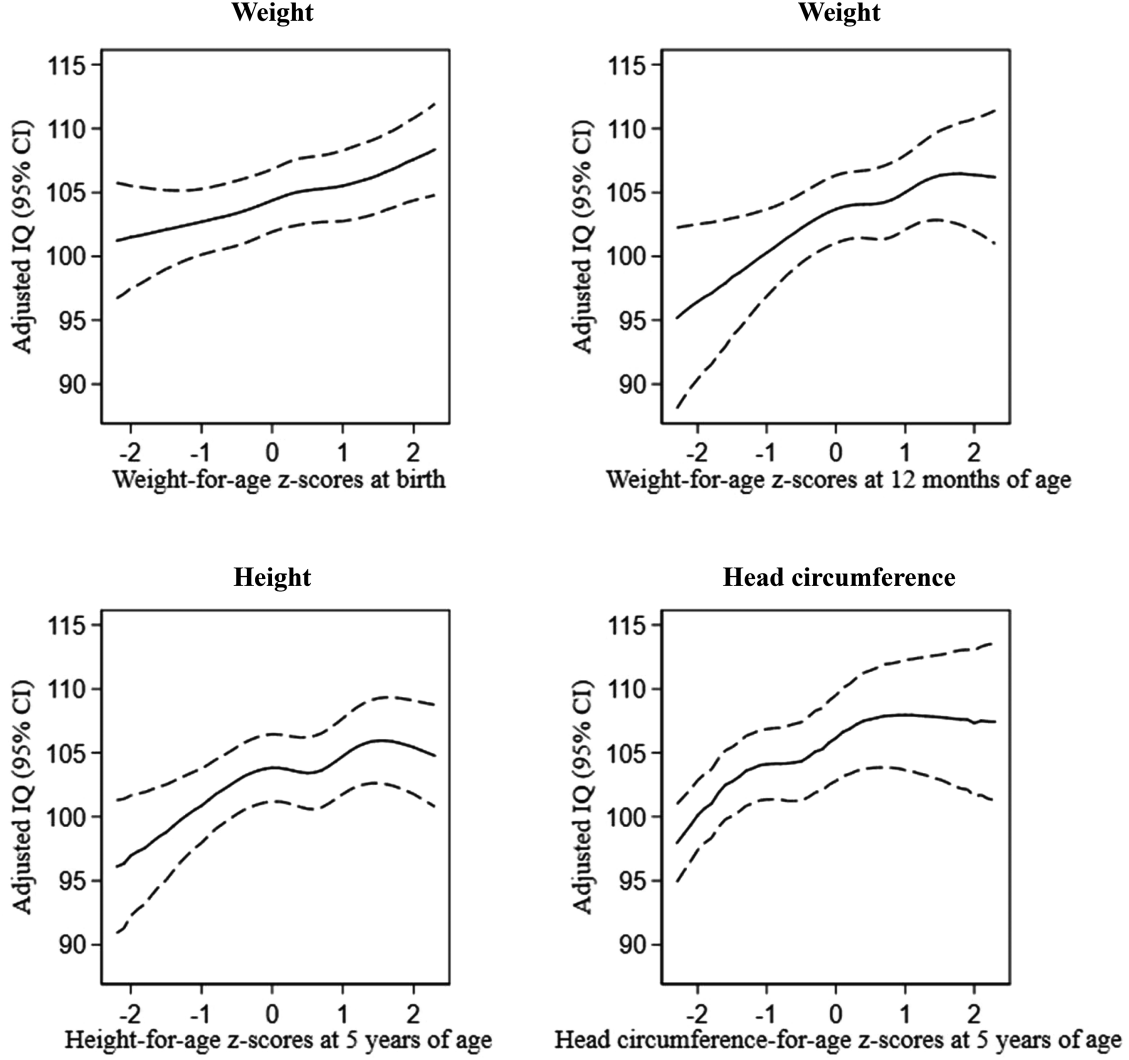
Restricted cubic splines of growth measures and IQ at 5 y of age. Adjusted for sex, socio-occupational status, birth order, gestational age, maternal age, maternal prepregnancy BMI, smoking, exercise and dietary intake during pregnancy, postpartum smoking, age when started in daycare, duration of any breastfeeding, time of introduction to solid food, paternal BMI 18 mo postpartum, maternal IQ, and the analyses of weight were further adjusted for length, and the analyses of height were further adjusted for weight, and finally the analyses of head circumference were further adjusted for weight and length. The splines are presented for a reference child using mean values and reference categories on covariates, see Table 1. IQ, intelligence quotient.

1 Indicates the reference groups used in the restricted cubic splines in Figure 2. For maternal age, BMI, IQ, paternal BMI, and gestational age the average value was used as the reference. IQ, intelligence quotient.

All statistical analyses were performed by using Stata/SE 15 (StataCorp).

## Results

Table 1 presents characteristics of the study population (*n* = 1719). The average maternal age at conception was 30 y, most of the mothers had above average socio-occupational status, and few of the mothers and fathers were obese before pregnancy. Of the children, 52% were boys and 52% were firstborns. Average birth weight was 3630 ± 486 g, length was 52.5 ± 2.2 cm, and head circumference was 35.4 ± 1.6 cm. Seventy-five percent of participants were breastfed fully or partially for >20 wk, and 75% of participants were introduced to solid food at the age of 4–5 mo.

The average IQ score for boys was 103.8 ± 13.4, and for girls 107.7 ± 11.8. Total, indirect, and direct effects of weight-for-age, height-for-age, and head-circumference-for-age *z*-scores on IQ at the age of 5 y are presented in **Table 2**. Estimates were only slightly attenuated when going from model A to model B, which was further adjusted for other growth measures (weight adjusted for length/height, length/height adjusted for weight, and head circumference adjusted for weight plus length/height). Estimates from model B are presented below.

**TABLE 2:** Regression coefficients (95% CI) of IQ at 5 y of age according to birth size and growth in infancy and childhood among 1719 term-born children^1^

	Total		Indirect		Direct		Percentage of total		Total		Indirect		Direct		Percentage of total
	Model A^2^								Model B^3^						
Weight-for-age *z*-score															
WAZ birth	1.41	(0.71, 2.11)	0.36	(0.01, 0.71)	1.06	(0.29, 1.82)	74.6		1.22	(0.50, 1.94)	0.14	(−0.05, 0.33)	1.08	(0.32, 1.84)	88.2
WAZ 5 mo	0.74	(0.03, 1.46)	1.15	(0.24, 2.07)	−0.41	(−1.55, 0.74)	26.3		0.59	(−0.16, 1.34)	0.95	(0.08, 1.82)	−0.36	(−1.52, 0.80)	27.4
WAZ 12 mo	1.61	(0.34, 2.89)	−0.20	(−0.85, 0.45)	1.82	(0.23, 3.40)	90.1		1.53	(0.14, 2.92)	−0.04	(−0.38, 0.29)	1.57	(0.01, 3.13)	97.4
WAZ 5 y	−0.30	(−1.28, 0.68)		NA		−0.30	(−1.28, 0.68)		−0.12	(−1.07, 0.83)		NA	−0.12	(−1.07, 0.83)	
Height-for-age *z*-score															
HAZ birth	0.72	(0.07, 1.38)	0.32	(−0.05, 0.70)	0.40	(−0.32, 1.12)	55.6		0.52	(−0.15, 1.19)	0.17	(−0.08, 0.42)	0.35	(−0.36, 1.07)	68.1
HAZ 5 mo	0.55	(−0.08, 1.19)	0.23	(−0.23, 0.70)	0.33	(−0.42, 1.08)	58.9		0.44	(−0.22, 1.10)	0.04	(−0.33, 0.40)	0.40	(−0.36, 1.16)	91.5
HAZ 12 mo	0.35	(−0.36, 1.07)	0.46	(0.06, 0.85)	−0.10	(−0.94, 0.75)	17.9		0.08	(−0.69, 0.84)	0.23	(0.04, 0.43)	−0.15	(−1.00, 0.69)	39.8
HAZ 5 y	0.97	(0.14, 1.80)		NA	0.97	(0.14, 1.80)			0.98	(0.17, 1.79)		NA	0.98	(0.17, 1.79)	
Head-circumference-for-age *z*-score															
HCAZ birth	0.52	(0.02, 1.02)	0.11	(−0.29, 0.51)	0.41	(−0.18, 1.00)	78.8		0.41	(−0.09, 0.91)	0.08	(−0.25, 0.40)	0.33	(−0.25, 0.91)	81.0
HCAZ 5 mo	0.23	(−0.65, 1.12)	1.36	(0.15, 2.57)	−1.13	(−2.74, 0.48)	45.4		0.22	(−0.69, 1.13)	1.18	(−0.03, 2.39)	−0.97	(−2.61, 0.68)	45.0
HCAZ 12 mo	1.87	(0.21, 3.53)	1.75	(0.74, 2.76)	0.12	(−2.06, 2.29)	6.4		1.73	(−0.05, 3.50)	1.39	(0.50, 2.27)	0.34	(−1.91, 2.59)	19.8
HCAZ 5 y	2.36	(1.02, 3.71)		NA	2.36	(1.02, 3.71)			2.09	(0.78, 3.41)		NA	2.09	(0.78, 3.41)	

1 In the path model all growth measures were added to the model separately, but because they are conditioned on previous growth measures, a greater WAZ at 12 mo, for example, should be interpreted as greater gain in WAZ from 5 to 12 mo. HAZ, length/height-for-age *z*-score; HCAZ, head-circumference-for-age *z*-score; IQ, intelligence quotient; WAZ: weight-for-age *z*-score.

2 Adjusted for sex, gestational age, socio-occupational status, birth order, maternal age, maternal prepregnancy BMI; and during pregnancy, smoking, exercise, and dietary intake; and postpartum, smoking, age when started in daycare, duration of any breastfeeding, time of introduction to solid food, paternal BMI 18 mo postpartum, and maternal IQ.

3 Adjusted for all model A factors plus height-for-age at each time point for the analyses of weight-for-age, weight-for-age at each time point for the analyses of height-for-age, and weight-for-age and height-for-age at each time point for the analyses of head-circumference-for-age.

We observed that greater weight at birth was associated with higher IQ in childhood; per *z*-score increase, the IQ score increased by 1.22 (95% CI: 0.50, 1.94) points. Also, length and head circumference at birth were positively but nonsignificantly associated with IQ in childhood. The effects of all 3 growth measures on IQ were mainly direct (68–88% of the effect was direct), suggesting an association of birth size not mediated through later growth.

Increasing weight gain in early infancy was indirectly associated with higher IQ, thus mediated through greater weight at 12 mo. Also, weight gain in late infancy was positively associated with IQ; however, this association was direct. This means that it was not mediated through a subsequent greater weight at 5 y. In contrast, gain in length and head circumference in late infancy were mainly observed to be indirectly associated with IQ, meaning that the positive effects were mediated through a greater height and head circumference at 5 y.

For growth in childhood, we observed that increased growth in height and growth in head circumference were associated with a higher IQ. Thus, per *z*-score increase in height and head circumference at 5 y, the IQ was 0.98 (95% CI: 0.17, 1.79) and 2.09 (95% CI: 0.78, 3.41) points higher, respectively. This supported the previously mentioned indirect effect of length and head circumference in late infancy mediated through greater height and head circumference in childhood. In contrast, greater weight gain in childhood from 12 mo to 5 y seemed not to be associated with higher IQ in childhood. Instead, the estimate indicated a weak negative association with IQ [−0.12 (95% CI: −1.07, 0.83)].

Restricted cubic splines (**Figure 2**) showed a positive association of birth weight, and there seemed to be no threshold, thus increasing birth weight was associated with greater IQ throughout the birth weight range. However, the positive association between greater weight in late infancy at 12 mo and IQ seemed to level off around 1.5 *z*-scores. Similarly, the positive association between greater height and head circumference at 5 y and IQ, adjusted for previous growth measurements, also leveled off around 1 to 1.5 *z*-scores.

## Discussion

In children born at term in a high-income country, we observed that both prenatal and postnatal growth were associated with positive increments in childhood IQ. Greater size at birth, especially birth weight, was positively related to IQ, independent of later growth. Also, growth in infancy, especially weight gain and growth in head circumference from 5 to 12 mo of age, were positively associated with IQ. In the childhood period, after adjusting for previous growth, we found both greater gain in height and in head circumference between 12 mo and 5 y to be positively related to IQ, whereas greater weight gain in the same period seemed not to be associated with IQ.

Others have observed a positive association between birth weight and childhood IQ (17, 18), and 1 study, which also used structural equation modeling, found that the potential effect of birth weight was mainly direct (41). Results from previous studies on the influence of weight gain in childhood do not agree. Some observed that weight gain until 4–7 y of age was positively associated with childhood IQ (17, 18), others observed no influence of weight gain beyond 1 y of age (20), and some observed a small negative effect of weight gain from birth throughout the first 10 y of life (41). None of these studies tried to separate weight gain from length/height as we did. Greater weight for a given length/height might be a proxy of adiposity/increasing fat mass. In a recent study, researchers found that gain in weight-for-height from 1 to 2 y postpartum was possibly negatively associated with IQ at 5–8 y (42), indicating that early-life adiposity could affect neurodevelopment. We did not measure weight at 2 y, so our measure of childhood weight gain covers weight gain from 1 to 5 y of age, and our findings indicate that greater weight conditioned on height in childhood could be associated with lower IQ. In infancy, we observed that weight gain was positively associated with IQ; however, this beneficial association should of course be weighed against the potential negative associations between rapid weight gain in early infancy and greater risk of obesity and higher blood pressure in childhood that others have observed (43, 44). For growth in height, we showed a positive association between increasing height in childhood and IQ, which has also been observed by others (17, 41, 45, 46). Some also found that greater length at birth and greater length in infancy were associated with better childhood IQ (18). We found a positive association for length at birth only when we did not adjust for birth weight, thus part of the observed effect of birth length could be due to a greater birth weight. A potential mechanism linking childhood height with IQ is suggested to be through greater gray and white matter volumes of the brain, because these have been shown to be positively correlated with both height and IQ in children aged 5–18 y (47). Head circumference is shown to be a reliable estimate of brain volume in young children (48). Our findings are supported by some, who also observed postnatal growth in head circumference to be more important than prenatal growth in determining childhood IQ (49), but not by others, who found growth in head circumference during the first year to be the most important (19, 20). Taken together, the existing evidence and our findings suggest that among healthy children born at term, growth is important in relation to childhood IQ. However, the evidence is inconclusive with respect to the relative importance of the different time periods. This might be partly explained by different measuring methods, different time periods, and different sample sizes.

The children in our study population were all living in a high-income country with taxpayer-funded access to health care, and most of them had mothers with above average socio-occupational status. Thus, it is likely that they did not suffer from undernutrition or severe and prolonged infectious diseases affecting growth (50). However, we still observed differences in growth that apparently affected childhood IQ; thus other social or biological mechanisms might be present. Insulin-like growth factor I (IGF-I) is a key factor in somatic growth regulation because it mediates the effect of growth hormones and is hypothesized to mediate brain development in children. A positive association has been observed between IGF-I levels and IQ in children aged 7–8 y (51). In addition, a twin study suggested that the same genetic factors could affect both growth and IQ (52). More research is needed to clarify the mechanisms that could drive these associations in a well-nourished population of children born at term, growing up in a country with a high-quality health care system.

Strengths of our study include the longitudinal design and the ability to include the 3 growth measures, weight, length/height, and head circumference, at all time points. This allowed us to examine if change in growth from birth throughout the first 5 y of life was related to childhood IQ. Also, by separating the different growth measures, we were able to study their independent contribution. We applied a statistical approach that provided insight into potential direct and indirect pathways between prenatal and postnatal growth and childhood IQ. Moreover, the present study is a substudy within the DNBC, which contains extensive information on several potential confounders including breastfeeding (53) and maternal socio-occupational status and IQ, which could be strongly associated with offspring IQ and affect growth as well.

Our study also has some limitations that should be addressed. Although growth parameters were measured by health professionals, measurement errors are inevitable, perhaps more for length and head circumference than for weight, and perhaps more for length at birth/infancy than height in childhood. We would expect such measurement errors to be unrelated to the outcome, potentially attenuating the associations. Further, childhood growth was defined as growth from 12 mo to 5 y, which is a long period that could hide important variation, and ≥1 measurements in that period would have provided more detailed information on influence of growth in specific periods of childhood. Despite the fact that women of higher socioeconomic status were overrepresented in the DNBC (54), and our study population further was selected on participation in the LDPS, risk of selection bias need not to be a problem if selection was not related to the studied outcome. A previous study on selection bias within the DNBC has shown no or little bias on effect estimates within the cohort after adjustments (55). The oversampling in the LDPS of high alcohol intake is expected to reduce the power of the study but need not affect validity. Further, we cannot rule out that the limited power of the study might have caused some false-negative findings, and that multiple testing might have caused some false-positive findings. However, path analysis reduced the risk of false-positive findings compared with running each regression separately. Finally, even though we adjusted for many potential confounders, we cannot rule out unmeasured or residual confounding. Also, we cannot rule out the possibility of reverse causation, because 5-y growth and IQ were measured simultaneously. Greater height and head circumference could have led to better IQ, but better IQ could also have led to greater height and head circumference. It is possible that brain development and function in very early life could determine later growth and nutritional intake.

In conclusion, we observed that in children born at term with free access to health care, growth in utero and up until the age of 5 y was positively associated with childhood IQ. Greater birth size in itself was an indicator of higher IQ, with weight gain as the strongest growth parameter until 12 mo of age. After 12 mo until 5 y of age, growth in height and head circumference were most important. The effect sizes we observed could be important on a population level, and even small changes in a nation's IQ have been shown to influence human capital and economic living standards (56). Thus, we need strategies promoting optimal growth so that children throughout the world can reach their genetic growth and cognitive potential through optimal nutrition and access to high-quality health care and education.

## Supplementary Material

nqaa051_Supplement_File

## Notes

The Danish National Birth Cohort (DNBC) was established with a significant grant from the Danish National Research Foundation. Additional support was obtained from the Danish Regional Committees, the Pharmacy Foundation, the Egmont Foundation, the March of Dimes Birth Defects Foundation, the Health Foundation, and other minor grants. The DNBC Biobank has been supported by the Novo Nordisk Foundation and the Lundbeck Foundation. Follow-up of mothers and children has been supported by the Danish Medical Research Council (SSVF 0646, 271-08-0839/06-066023, O602-01042B, 0602–02738B), the Lundbeck Foundation (195/04, R100-A9193), the Innovation Fund Denmark 0603-00294B (09-067124), the Nordea Foundation (02-2013-2014), Aarhus Ideas (AU R9-A959-13-S804), University of Copenhagen Strategic Grant (IFSV 2012), and the Danish Council for Independent Research (DFF-4183-00594 and DFF-4183-00152). The Lifestyle During Pregnancy Study was supported primarily by the CDC, Atlanta, GA, USA. The present study was supported by the Bill & Melinda Gates Foundation (OPP1123866).

The article's contents are the sole responsibility of the authors and may not necessarily represent the official views of the Bill & Melinda Gates Foundation or other agencies that may have supported the present study. The data on which the findings of this study are based came from the DNBC, and restrictions apply to these data, which were used under license for the current study and are not publicly available. The DNBC welcomes requests for data, which must include a short protocol with a specific research question and a plan for analysis. More information can be found on www.dnbc.dk.

Supplemental Figures 1 and 2 and Supplemental Table 1 are available from the “Supplementary data” link in the online posting of the article and from the same link in the online table of contents at https://academic.oup.com/ajcn/.

Abbreviations used: DNBC, Danish National Birth Cohort; GP, general practitioner; HAZ, height-for-age *z*-score; HCAZ, head-circumference-for-age *z*-score; IGF-I, insulin-like growth factor I; IQ, intelligence quotient; LDPS, Lifestyle During Pregnancy Study; MBR, Medical Birth Registry; WAZ, weight-for-age *z*-score; WPPSI-R, Wechsler Primary and Preschool Scales of Intelligence–Revised.

## References

[1] Morgan BL Nutritional requirements for normative development of the brain and behavior. Ann NY Acad Sci. 1990;602:127–32. 1700659 10.1111/j.1749-6632.1990.tb22734.x

[2] Shenkin SD, Starr JM, Deary IJ Birth weight and cognitive ability in childhood: a systematic review. Psychol Bull. 2004;130:989–1013. 15535745 10.1037/0033-2909.130.6.989

[3] Flensborg-Madsen T, Mortensen EL Birth weight and intelligence in young adulthood and midlife. Pediatrics. 2017;139:e20163161. 28562263 10.1542/peds.2016-3161

[4] Sørensen HT, Sabroe S, Olsen J, Rothman KJ, Gillman MW, Fischer P Birth weight and cognitive function in young adult life: historical cohort study. BMJ. 1997;315:401–3. 9277604 10.1136/bmj.315.7105.401 PMC2127280

[5] Mangin KS, Horwood LJ, Woodward LJ Cognitive development trajectories of very preterm and typically developing children. Child Dev. 2017;88:282–98. 27364183 10.1111/cdev.12585

[6] Martínez-Cruz CF, Poblano A, Fernández-Carrocera LA, Jiménez-Quiróz R, Tuyú-Torres N Association between intelligence quotient scores and extremely low birth weight in school-age children. Arch Med Res. 2006;37:639–45. 16740436 10.1016/j.arcmed.2005.12.001

[7] Casey PH, Whiteside-Mansell L, Barrett K, Bradley RH, Gargus R Impact of prenatal and/or postnatal growth problems in low birth weight preterm infants on school-age outcomes: an 8-year longitudinal evaluation. Pediatrics. 2006;118:1078–86. 16951001 10.1542/peds.2006-0361

[8] Taine M, Charles M-A, Beltrand J, Rozé JC, Léger J, Botton J, Heude B Early postnatal growth and neurodevelopment in children born moderately preterm or small for gestational age at term: a systematic review. Paediatr Perinat Epidemiol. 2018;32:268–80. 29691880 10.1111/ppe.12468

[9] Sammallahti S, Heinonen K, Andersson S, Lahti M, Pirkola S, Lahti J, Pesonen A-K, Lano A, Wolke D, Eriksson JG et al. Growth after late-preterm birth and adult cognitive, academic, and mental health outcomes. Pediatr Res. 2017;81:767–74. 28056012 10.1038/pr.2016.276

[10] Varella MH, Moss WJ Early growth patterns are associated with intelligence quotient scores in children born small-for-gestational age. Early Hum Dev. 2015;91:491–7. 26100090 10.1016/j.earlhumdev.2015.06.002

[11] Lei X, Chen Y, Ye J, Ouyang F, Jiang F, Zhang J The optimal postnatal growth trajectory for term small for gestational age babies: a prospective cohort study. J Pediatr. 2015;166:54–8. 25444014 10.1016/j.jpeds.2014.09.025

[12] Wang P-W, Fang L-J, Tsou K-I, Taiwan Infant Developmental Collaborative Study Group The growth of very-low-birth-weight infants at 5 years old in Taiwan. Pediatr Neonatol. 2014;55:114–19. 24126010 10.1016/j.pedneo.2013.08.001

[13] Matte TD, Bresnahan M, Begg MD, Susser E Influence of variation in birth weight within normal range and within sibships on IQ at age 7 years: cohort study. BMJ. 2001;323:310–14. 11498487 10.1136/bmj.323.7308.310 PMC37317

[14] Tong S, Baghurst P, McMichael A Birthweight and cognitive development during childhood. J Paediatr Child Health. 2006;42:98–103. 16509907 10.1111/j.1440-1754.2006.00805.x

[15] Broekman BFP, Chan Y-H, Chong Y-S, Quek S-C, Fung D, Low Y-L, Ooi Y-P, Gluckman PD, Meaney MJ, Wong T-Y et al. The influence of birth size on intelligence in healthy children. Pediatrics. 2009;123:e1011–16. 19482733 10.1542/peds.2008-3344

[16] Smithers LG, Lynch JW, Yang S, Dahhou M, Kramer MS Impact of neonatal growth on IQ and behavior at early school age. Pediatrics. 2013;132:e53–60. 23776123 10.1542/peds.2012-3497 PMC4530288

[17] Huang C, Martorell R, Ren A, Li Z Cognition and behavioural development in early childhood: the role of birth weight and postnatal growth. Int J Epidemiol. 2013;42:160–71. 23243117 10.1093/ije/dys207 PMC3600622

[18] Yang S, Tilling K, Martin R, Davies N, Ben-Shlomo Y, Kramer MS Pre-natal and post-natal growth trajectories and childhood cognitive ability and mental health. Int J Epidemiol. 2011;40:1215–26. 21764769 10.1093/ije/dyr094

[19] Gale CR, O'Callaghan FJ, Bredow M, Martyn CN, Avon Longitudinal Study of Parents and Children Study Team. The influence of head growth in fetal life, infancy, and childhood on intelligence at the ages of 4 and 8 years. Pediatrics. 2006;118:1486–92. 17015539 10.1542/peds.2005-2629

[20] Pongcharoen T, Ramakrishnan U, DiGirolamo AM, Winichagoon P, Flores R, Singkhornard J, Martorell R Influence of prenatal and postnatal growth on intellectual functioning in school-aged children. Arch Pediatr Adolesc Med. 2012;166:411–16. 22566539 10.1001/archpediatrics.2011.1413

[21] Beyerlein A, Ness AR, Streuling I, Hadders-Algra M, von Kries R Early rapid growth: no association with later cognitive functions in children born not small for gestational age. Am J Clin Nutr. 2010;92:585–93. 20592132 10.3945/ajcn.2009.29116

[22] Georgiadis A, Benny L, Crookston BT, Duc LT, Hermida P, Mani S, Woldehanna T, Stein AD, Behrman JR Growth trajectories from conception through middle childhood and cognitive achievement at age 8 years: evidence from four low- and middle-income countries. SSM Popul Health. 2016;2:43–54. 27110590 10.1016/j.ssmph.2016.01.003 PMC4838904

[23] Olsen J, Melbye M, Olsen SF, Sorensen TI, Aaby P, Andersen AM, Taxbol D, Hansen KD, Juhl M, Schow TB et al. The Danish National Birth Cohort—its background, structure and aim. Scand J Public Health. 2001;29:300–7. 11775787 10.1177/14034948010290040201

[24] Kesmodel US, Underbjerg M, Kilburn TR, Bakketeig L, Mortensen EL, Landrø NI, Schendel D, Bertrand J, Grove J, Ebrahim S et al. Lifestyle during pregnancy: neurodevelopmental effects at 5 years of age. The design and implementation of a prospective follow-up study. Scand J Public Health. 2010;38:208–19. 20064917 10.1177/1403494809357093

[25] Eriksen H-LF, Kesmodel US, Underbjerg M, Kilburn TR, Bertrand J, Mortensen EL Predictors of intelligence at the age of 5: family, pregnancy and birth characteristics, postnatal influences, and postnatal growth. PLoS One. 2013;8:e79200. 24236109 10.1371/journal.pone.0079200 PMC3827334

[26] Andersen AM, Olsen J. The Danish National Birth Cohort: selected scientific contributions within perinatal epidemiology and future perspectives. Scand J Public Health. 2011;39:115–20. 21775368 10.1177/1403494811407674

[27] Vidmar SI, Cole TJ, Pan H Standardizing anthropometric measures in children and adolescents with functions for egen: update. Stata J. 2013;13:366–78.

[28] Wechsler D Manual for the Wechsler Preschool and Primary Scale of Intelligence. Revised UK edition. Sidcup (Kent): The Psychological Corporation; 1990.

[29] Hurks P, Hendriksen J, Dek J, Kooij A Accuracy of short forms of the Dutch Wechsler Preschool and Primary Scale of Intelligence: third edition. Assessment. 2016;23:240–9. 25804438 10.1177/1073191115577189

[30] Wechsler D Manual for the Wechsler Preschool and Primary Scale of Intelligence – revised. Swedish version. Stockholm: Psykologiförlaget AB; 1999.

[31] Nohr EA, Bech BH, Davies MJ, Frydenberg M, Henriksen TB, Olsen J Prepregnancy obesity and fetal death: a study within the Danish National Birth Cohort. Obstet Gynecol. 2005;106:250–9. 16055572 10.1097/01.AOG.0000172422.81496.57

[32] Knudsen VK, Heitmann BL, Halldorsson TI, Sorensen TI, Olsen SF Maternal dietary glycaemic load during pregnancy and gestational weight gain, birth weight and postpartum weight retention: a study within the Danish National Birth Cohort. Br J Nutr. 2013;109:1471–8. 22906835 10.1017/S0007114512003443

[33] Wechsler D Manual for the Wechsler Adult Intelligence Scale. New York: The Psychological Corporation; 1955.

[34] Raven J Standard progressive matrices. Oxford: Oxford Psychologists Press; 1958.

[35] Kesmodel US, Eriksen H-LF, Underbjerg M, Kilburn TR, Støvring H, Wimberley T, Mortensen EL The effect of alcohol binge drinking in early pregnancy on general intelligence in children. BJOG. 2012;119:1222–31. 22712770 10.1111/j.1471-0528.2012.03395.x

[36] Gamborg M, Andersen PK, Baker JL, Budtz-Jorgensen E, Jorgensen T, Jensen G, Sorensen TI Life course path analysis of birth weight, childhood growth, and adult systolic blood pressure. Am J Epidemiol. 2009;169:1167–78. 19357327 10.1093/aje/kwp047 PMC2732973

[37] StataCorp. Stata structural equation modeling reference manual release 15. College Station (TX): StataCorp; 2017.

[38] Dong Y, Peng C-YJ Principled missing data methods for researchers. SpringerPlus. 2013;2:222. 23853744 10.1186/2193-1801-2-222 PMC3701793

[39] van der Heijden GJ, Donders AR, Stijnen T, Moons KG Imputation of missing values is superior to complete case analysis and the missing-indicator method in multivariable diagnostic research: a clinical example. J Clin Epidemiol. 2006;59:1102–9. 16980151 10.1016/j.jclinepi.2006.01.015

[40] Sterne JA, White IR, Carlin JB, Spratt M, Royston P, Kenward MG, Wood AM, Carpenter JR Multiple imputation for missing data in epidemiological and clinical research: potential and pitfalls. BMJ. 2009;338:b2393. 19564179 10.1136/bmj.b2393 PMC2714692

[41] Silva A, Metha Z, O'Callaghan FJ The relative effect of size at birth, postnatal growth and social factors on cognitive function in late childhood. Ann Epidemiol. 2006;16:469–76. 16165368 10.1016/j.annepidem.2005.06.056

[42] Li N, Yolton K, Lanphear BP, Chen A, Kalkwarf HJ, Braun JM Impact of early-life weight status on cognitive abilities in children. Obesity (Silver Spring, Md). 2018;26:1088–95. 10.1002/oby.22192 PMC5975980 29797555

[43] Perng W, Rifas-Shiman SL, Kramer MS, Haugaard LK, Oken E, Gillman MW, Belfort MB Early weight gain, linear growth, and mid-childhood blood pressure: a prospective study in project viva. Hypertension. 2016;67:301–8. 26644238 10.1161/HYPERTENSIONAHA.115.06635 PMC4769100

[44] Rotevatn TA, Overgaard C, Melendez-Torres GJ, Mortensen RN, Ullits LR, Høstgaard AMB, Torp-Pedersen C, Bøggild H Infancy weight gain, parental socioeconomic position, and childhood overweight and obesity: a Danish register-based cohort study. BMC Public Health. 2019;19:1209. 31477065 10.1186/s12889-019-7537-z PMC6720844

[45] Richards M, Hardy R, Kuh D, Wadsworth MEJ Birthweight, postnatal growth and cognitive function in a national UK birth cohort. Int J Epidemiol. 2002;31:342–8. 11980795

[46] Pearce MS, Deary IJ, Young AH, Parker L Growth in early life and childhood IQ at age 11 years: the Newcastle Thousand Families Study. Int J Epidemiol. 2005;34:673–7. 15746206 10.1093/ije/dyi038

[47] Taki Y, Hashizume H, Sassa Y, Takeuchi H, Asano M, Asano K, Kotozaki Y, Nouchi R, Wu K, Fukuda H et al. Correlation among body height, intelligence, and brain gray matter volume in healthy children. Neuroimage. 2012;59:1023–7. 21930215 10.1016/j.neuroimage.2011.08.092

[48] Bartholomeusz HH, Courchesne E, Karns CM Relationship between head circumference and brain volume in healthy normal toddlers, children, and adults. Neuropediatrics. 2002;33:239–41. 12536365 10.1055/s-2002-36735

[49] Gale CR, O'Callaghan FJ, Godfrey KM, Law CM, Martyn CN Critical periods of brain growth and cognitive function in children. Brain J Neurol. 2004;127:321–9. 10.1093/brain/awh034 14645144

[50] Black RE Patterns of growth in early childhood and infectious disease and nutritional determinants. Nestle Nutr Inst Workshop Ser. 2017;87:63–72. 28315889 10.1159/000448938

[51] Gunnell D, Miller LL, Rogers I, Holly JMP, ALSPAC Study Team Association of insulin-like growth factor I and insulin-like growth factor-binding protein-3 with intelligence quotient among 8- to 9-year-old children in the Avon Longitudinal Study of Parents and Children. Pediatrics. 2005;116:e681–6. 16263982 10.1542/peds.2004-2390

[52] Silventoinen K, Iacono WG, Krueger R, McGue M Genetic and environmental contributions to the association between anthropometric measures and IQ: a study of Minnesota twins at age 11 and 17. Behav Genet. 2012;42:393–401. 22139438 10.1007/s10519-011-9521-y PMC3297715

[53] Strøm M, Mortensen EL, Kesmodel US, Halldorsson T, Olsen J, Olsen SF Is breast feeding associated with offspring IQ at age 5? Findings from prospective cohort: Lifestyle During Pregnancy Study. BMJ Open. 2019;9:e023134. 10.1136/bmjopen-2018-023134 PMC6549733 31152024

[54] Jacobsen TN, Nohr EA, Frydenberg M Selection by socioeconomic factors into the Danish National Birth Cohort. Eur J Epidemiol. 2010;25:349–55. 20349116 10.1007/s10654-010-9448-2

[55] Nohr EA, Frydenberg M, Henriksen TB, Olsen J Does low participation in cohort studies induce bias?. Epidemiol Camb Mass. 2006;17:413–18. 10.1097/01.ede.0000220549.14177.60 16755269

[56] Jones G, Schneider WJ Intelligence, human capital, and economic growth: a Bayesian averaging of classical estimates (BACE) approach. J Econ Growth. 2006;11:71–93.

