# Supplementary material for: Associations of birth size, infancy, and childhood growth with intelligence quotient at 5 years of age: a Danish cohort study
Source: Am J Clin Nutr. 2020 Mar 31;112(1):96–105. doi: 10.1093/ajcn/nqaa051 (PMC7326594; doi:10.1093/ajcn/nqaa051)
Supplement: nqaa051_Supplement_File [file nqaa051_supplement_file.docx]

**Online-Supplementary Material**

**Associations of birth size, infancy and childhood growth with Intelligence Quotient (IQ) at 5 years of age: a Danish cohort study**

Helene Kirkegaard, Sören Möller, Chunsen Wu, Jonas Häggström, Sjurdur Frodi Olsen, Jørn Olsen, Ellen Aagaard Nohr

**Affiliations:**

Research Unit of Obstetrics and Gynecology, Department of Clinical Research, University of Southern Denmark, Kløvervænget 10, 10th floor, 5000 Odense C, Denmark (HK, CW, EAN)

Open Patient Data Explorative Network (OPEN), Department of Clinical Research, University of Southern Denmark and Odense University Hospital, J.B. Winsløws Vej 9a 3rd floor, 5000 Odense C, Denmark (SM, HK)

MTEK Sciences Inc., Vancouver, British Columbia (BC), V5Z 1J5, Canada (JH)

Department of Epidemiology Research, Statens Serum Institute, Artillerivej 5, 206/308, 2300 Copenhagen S, Denmark (SFO)

Department of Clinical Epidemiology, Aarhus University Hospital, Olof Palmes Allé 43-45, 8200 Aarhus N, Denmark (JO)


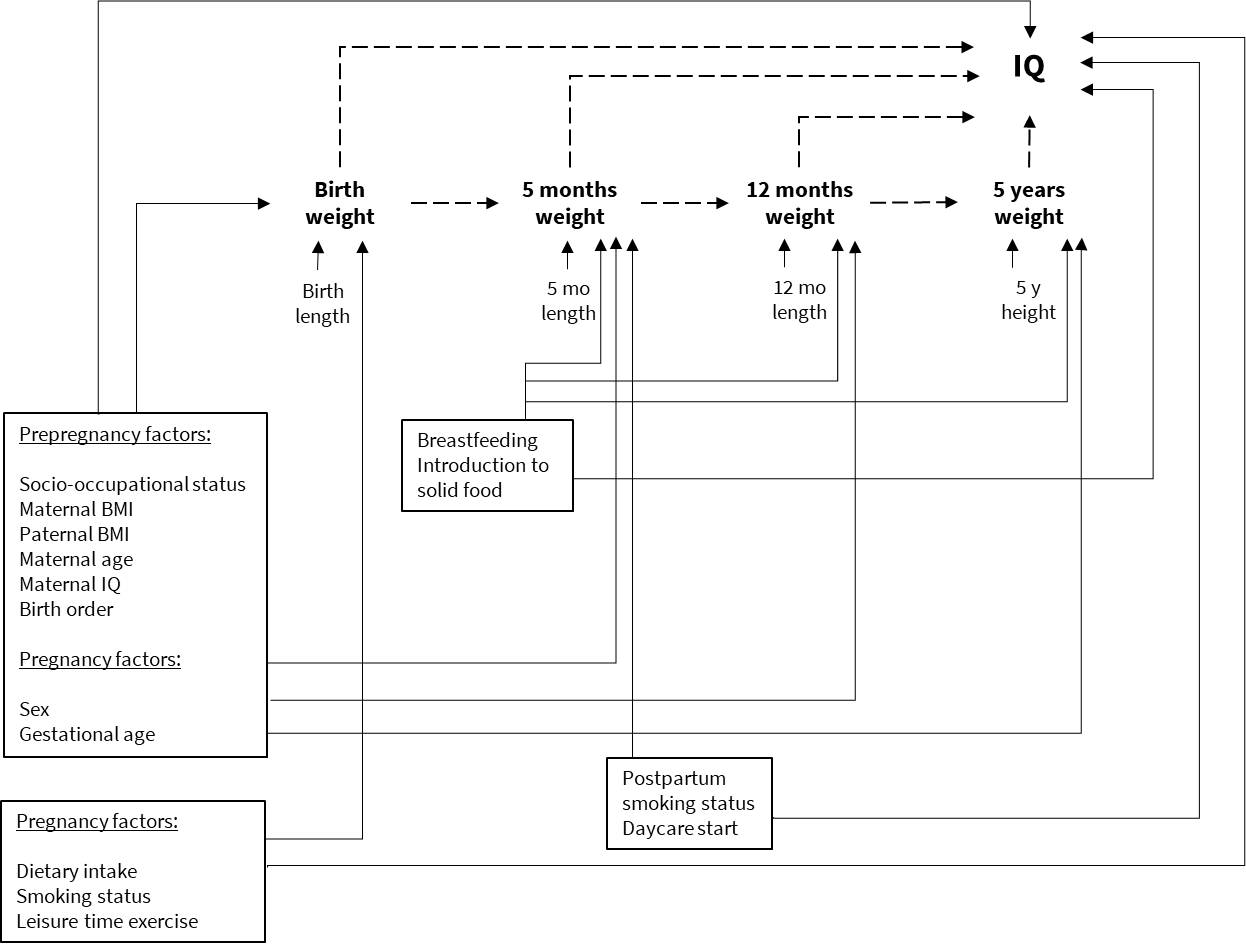


**Supplemental Figure 1.** Direct acyclic graph for the path model of weight as an example. Models of length/height and head circumference was also estimated. The dotted lines present the research question and the full lines the covariates adjusted for.

IQ: Intelligence Quotient; mo: months; y: years

Weight-for-age path analysis


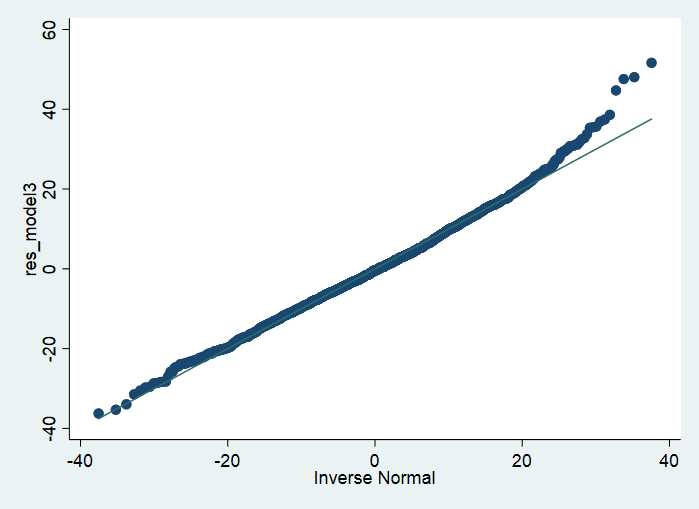


Length/height-for-age path analysis


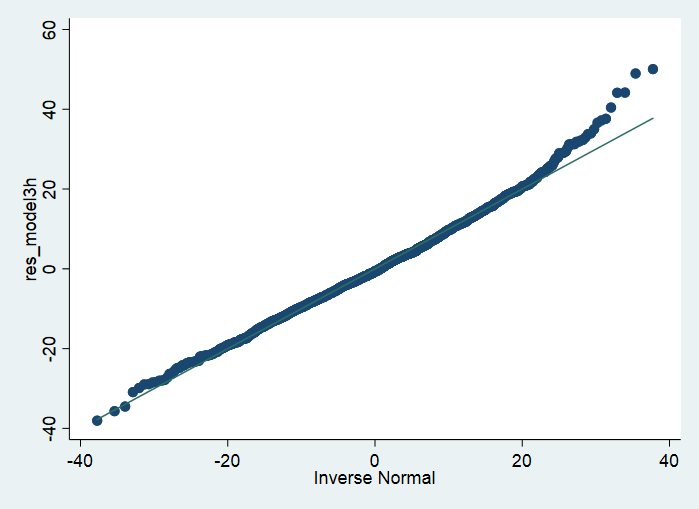


Head circumference-for-age path analysis


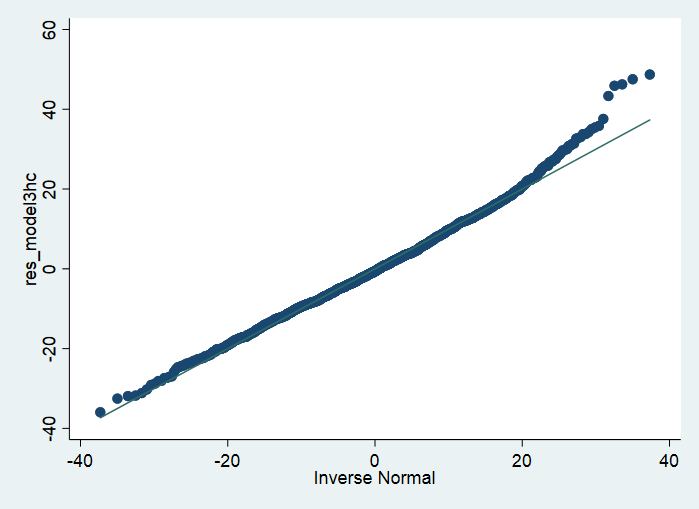


**Supplemental Figure 2.** Residual plots from path analyses of weight-for-age, length/height-for-age, and head circumference-for-age z-score at birth, 5 months, 12 months and 5 years of age among 1,719 term-born children.

| **Supplemental Table 1.** Regression coefficients of IQ at 5 years of age according to birth size and growth in infancy and childhood until 5 years of age among 1,719 term-born children^a^ | | |
| --- | --- | --- |
|  | **Total** | |
|  | *β* | 95% CI |
| *Weight-for-age z score* |  |  |
| WAZ birth | 1.50 | (0.79, 2.21) |
| WAZ 5 months | 0.52 | (-0.28, 1.32) |
| WAZ 12 months | 1.23 | (-0.15, 2.62) |
| WAZ 5 years | -0.82 | (-1.84, 0.21) |
| *Height-for-age z score* |  |  |
| HAZ birth | 0.97 | (0.27, 1.68) |
| HAZ 5 months | 0.08 | (-0.61, 0.76) |
| HAZ 12 months | 0.16 | (-0.63, 0.94) |
| HAZ 5 years | 1.25 | (0.35, 2.16) |
| *Head circumference-for-age z score* | |  |
| HCAZ birth | 0.45 | (-0.08, 0.98) |
| HCAZ 5 months | 0.48 | (-0.43, 1.39) |
| HCAZ 12 months | 2.65 | (0.92, 4.37) |
| HCAZ 5 years | 2.30 | (0.87, 3.72) |
| IQ: Intelligence Quotient; CI: Confidence Interval; WAZ: weight-for-age z-scores; HAZ: length/height-for-age z-scores; HCAZ: head circumference-for-age z-scores | | |
| Bold font indicates a statistically significant estimate. | | |
| ^a^ Adjusted for sex and gestational age | | |
